# The SARS-CoV-2 protein ORF3a inhibits fusion of autophagosomes with lysosomes

**DOI:** 10.1038/s41421-021-00268-z

**Published:** 2021-05-04

**Authors:** Yabin Zhang, Hao Sun, Rongjuan Pei, Binli Mao, Zhenyu Zhao, Huihui Li, Yong Lin, Kefeng Lu

**Affiliations:** 1 Department of Neurosurgery, State Key Laboratory of Biotherapy, West China Hospital, Sichuan University and The Research Units of West China, Chinese Academy of Medical Sciences, Chengdu, China; 2 Center for Biosafety Mega-Science, Wuhan Institute of Virology, Chinese Academy of Sciences, Wuhan, China; 3 Key Laboratory of Molecular Biology of Infectious Diseases (Chinese Ministry of Education), Department of Infectious Diseases, The Second Affiliated Hospital, Institute for Viral Hepatitis, Chongqing Medical University, Chongqing, China; 4 West China Second University Hospital, State Key Laboratory of Biotherapy, Sichuan University, Chengdu, China

**Keywords:** Autophagy, Innate immunity

## Abstract

Severe acute respiratory syndrome coronavirus 2 (SARS-CoV-2) has caused the ongoing coronavirus disease 2019 pandemic. How SARS-CoV-2 regulates cellular responses to escape clearance by host cells is unknown. Autophagy is an intracellular lysosomal degradation pathway for the clearance of various cargoes, including viruses. Here, we systematically screened 28 viral proteins of SARS-CoV-2 and identified that ORF3a strongly inhibited autophagic flux by blocking the fusion of autophagosomes with lysosomes. ORF3a colocalized with lysosomes and interacted with VPS39, a component of the homotypic fusion and protein sorting (HOPS) complex. The ORF3a–VPS39 interaction prohibited the binding of HOPS with RAB7, which prevented the assembly of fusion machinery, leading to the accumulation of unfused autophagosomes. These results indicated the potential mechanism by which SARS-CoV-2 escapes degradation; that is, the virus interferes with autophagosome–lysosome fusion. Furthermore, our findings will facilitate strategies targeting autophagy for conferring potential protection against the spread of SARS-CoV-2.

## Introduction

The pandemic of coronavirus disease 2019 (COVID-19), caused by the most-recently emergent member of the coronavirus family, severe acute respiratory syndrome coronavirus 2 (SARS-CoV-2), has transitioned this relatively understudied group of viruses to a worldwide public health priority in a matter of months^1–4^. SARS-CoV-2, an enveloped, positive-sense, single-stranded RNA coronavirus, belongs to the beta-coronavirus family^4,5^. Severe cases of COVID-19 are associated with hyperinflammation, also known as cytokine storm syndrome^6–8^. Uncontrolled infection and replication of SARS-CoV-2 cause this rampant inflammatory response that results in acute respiratory distress syndrome and end-organ injury^9–12^. There is an urgent need to understand how SARS-CoV-2 regulates host cellular responses and hijacks host cells to escape degradation/clearance.

Autophagy is a highly conserved cellular pathway involving the formation of autophagosomes to deliver cargoes, including long-lived proteins, protein aggregates, organelles, and infected viruses or bacteria, to lysosomes for degradation^13–15^. Autophagy is a constitutive pathway that can be stimulated when cells are under stress, such as when they are undergoing starvation or infection by pathogens^16–19^. When autophagy is initiated, an isolated membrane structure called a phagophore is formed at a special region associated with the endoplasmic reticulum (ER), Golgi apparatus, and ER–Golgi intermediate compartment^20–22^. Phagophores nucleate and expand by integrating membranes from multiple sources such that they become sealed double-membrane autophagosomes that engulf various substances^23^. After closure, the mature autophagosomes move to, dock onto, and subsequently fuse with lysosomes. This fusion process is mediated by the N-ethylmaleimide sensitive factor attachment protein receptor (SNARE) proteins syntaxin-17, SNAP29, and VAMP8, as well as the homotypic fusion and protein sorting (HOPS) complex^24–27^. After fusion with lysosomes, the inner membrane layer and enclosed cargo contents in the autophagosomes are ultimately degraded by lysosomal-residing hydrolases^17,23,28,29^.

While the autophagic degradation pathway can lead to the degradation of viral components, viruses have acquired elaborate strategies to evade, counteract, and sometimes co-opt protective mechanisms of host cells^30–32^. Accumulating evidence has shown that viruses can regulate multiple steps of autophagy, and vice versa in the host, autophagy may also play a crucial role in the viral lifecycle including infection, replication, and/or secretion^33–37^.

As a newly emerged human coronavirus, SARS-CoV-2 has been found to manipulate autophagy in host cells. Gene expression, protein levels, and signaling of the autophagy pathway have been found to be perturbed in SARS-CoV-2-infected lung cells and in peripheral blood mononuclear cells of COVID-19 patients^38–41^. Nevertheless, the specific mechanisms by which SARS-CoV-2 modulates autophagy remain elusive. We sought to address this knowledge gap by systematically clarifying whether and how SARS-CoV-2-encoding proteins regulate autophagy.

## Results

### Global analysis of SARS-CoV-2 protein regulation on autophagy

**Fig. 1:**
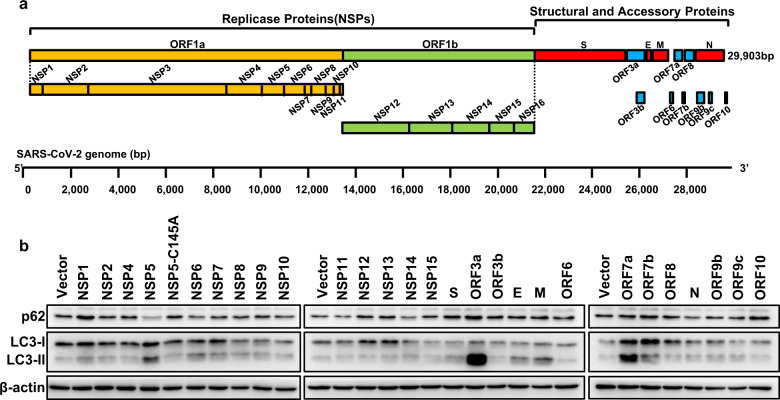
Global analysis of SARS-CoV-2-encoded proteins in autophagy. **a** Annotation of the SARS-CoV-2 genome and encoded proteins. **b** Screening of SARS-CoV-2-encoded proteins for the regulation of autophagy in HEK293T cells by determining the protein levels of p62 (substrate receptor) and LC3-II (autophagosome marker protein), which are both degraded by autophagy.

The 30-kb genome of SARS-CoV-2 encodes 14 open reading frames (ORFs) (Fig. 1a). ORF1a and ORF1b encode a polyprotein that is autoproteolytically processed into 16 nonstructural proteins (NSP1–16), forming the replicase/transcriptase complex (RTC) (Fig. 1a). The RTC contains papain-like protease NSP3, protease NSP5, primase complex NSP7–NSP8, RNA-dependent RNA polymerase NSP12, helicase/triphosphatase NSP13, exoribonuclease NSP14, endonuclease NSP15, and methyltransferases NSP10–NSP16^1,42^. At the 3′ end of the SARS-CoV-2 genome, 9 subgenomic RNAs encode 13 ORFs, including structural proteins: spike (S), envelope (E), membrane (M) and nucleocapsid (N), and 9 putative accessory factors (ORF3a, 3b, 6, 7a, 7b, 8, 9b, 9c, 10) (Fig. 1a). All proteins encoded by SARS-CoV-2 ORFs except for NSP3 and NSP16 were codon optimized and cloned into the mammalian expression vector pLVX-EF1alpha with a 2× Strep tag^39^. We then expressed these proteins in HEK293T cells to determine their effect on autophagy by detecting the protein levels of SQSTM1/p62 (a substrate receptor degraded by autophagy) and LC3-II (autophagosome membrane marker protein degraded through autophagy). The results showed that ORF3a caused a dramatical increase in p62 and LC3-II (Fig. 1b).

### SARS-CoV-2 ORF3a blocked autophagy and caused the accumulation of autophagosomes

**Fig. 2:**
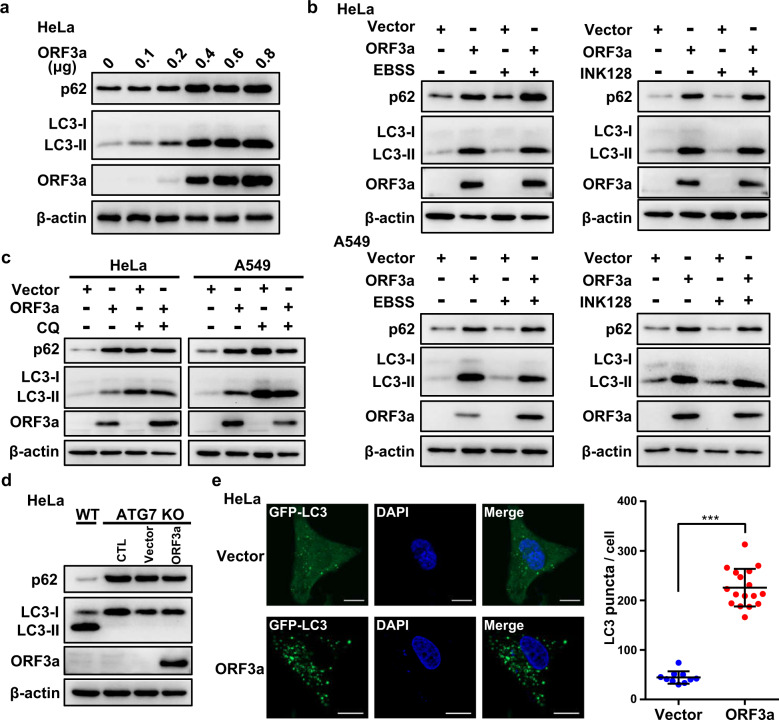
SARS-CoV-2 ORF3a blocked autophagy and caused the accumulation of autophagosomes. **a** ORF3a inhibited autophagy in a dose-dependent manner. Increasing amounts of SARS-CoV-2 ORF3a-expressing plasmids were transfected into HeLa cells, and the effect on autophagy was analyzed by detecting the protein levels of p62 and LC3-II. **b** ORF3a inhibited autophagy under autophagy-inducing conditions. ORF3a was expressed in HeLa or A549 cells with or without EBSS or INK128 treatment. The protein levels of p62 and LC3-II were then analyzed. **c** ORF3a cannot further inhibit autophagy under autophagy-blocking conditions. ORF3a was expressed in HeLa or A549 cells with or without subsequent treatment with CQ (chloroquine). The protein levels of p62 and LC3-II were then analyzed. **d** ORF3a cannot further inhibit autophagy in autophagy-blocked HeLa cells with the ATG7 gene knocked out. **e** ORF3 expression caused the accumulation of autophagosomes. ORF3a was expressed in HeLa cells with stable expression of GFP-LC3. Puncta of GFP-LC3 (representing autophagosomes) were observed and counted. Scale bars, 0.5 μm.

We then sought to confirm that the increase in p62 and LC3-II by ORF3a expression occurred by blocking autophagy. First, more human cell lines, including HeLa and A549 cells, were used to confirm the increase in p62 and LC3-II by ORF3a expression (Supplementary Fig. S1a). Increasing amounts of ORF3a caused elevated the accumulation of p62 and LC3-II proteins (Fig. 2a). A high level of ORF3a was detected in SARS-CoV-2 virus-infected human Calu-3 cells (Supplementary Fig. S1b), indicating that ORF3a inhibition of autophagy may occur in authentic SARS-CoV-2 infection. Furthermore, among the ORF3a homologs from α-CoV (HCoV-229E), β-CoV (SARS-CoV-2, SARS-CoV, and Middle East respiratory syndrome coronavirus (MERS-CoV)), and γ-CoV (IBV), SARS-CoV-2 ORF3a uniquely inhibited autophagy (Supplementary Fig. S1c). We then determined the effect of ORF3a under autophagy stimulation (short-term EBSS starvation or INK128 treatment) or autophagy blockade (chloroquine, CQ). The increase in p62 and LC3-II by ORF3a expression was retained when autophagy was stimulated (Fig. 2b). However, when autophagy was previously blocked by CQ, resulting in an increase in p62 and LC3-II, ORF3a did not cause a further increase (Fig. 2c). Similarly, when autophagy was blocked by ATG7-gene knockout, ORF3a did not induce further increases in p62 or LC3-II (Fig. 2d). These results indicated that ORF3 indeed blocked autophagy such that when autophagy was previously blocked by CQ or ATG7 knockout, its effect on autophagy was preempted. ORF3a may block autophagy by inhibiting the upstream steps (autophagosome formation) or by inhibiting the downstream steps (autophagosome fusion) of the autophagy process. In the former case, fewer autophagosomes would be formed in cells, while in the latter case, the accumulation of more autophagosomes would be evident. A dramatically increased number of autophagosomes (shown by GFP-LC3 puncta) was observed (Fig. 2e), which suggested that ORF3a may block autophagy by abolishing the fusion of autophagosomes with lysosomes, not by abolishing autophagosome formation.

### SARS-CoV-2 ORF3a blocked the fusion of autophagosomes with lysosomes

**Fig. 3:**
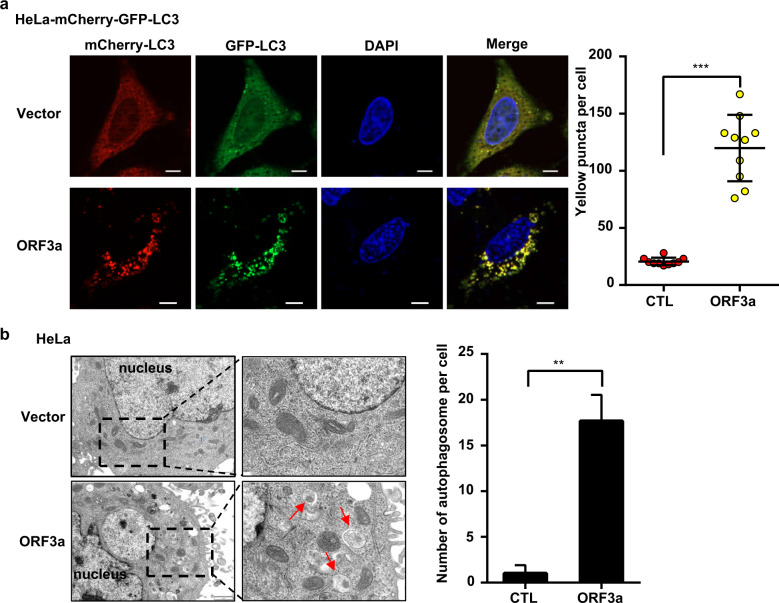
ORF3a blocked autophagosome–lysosome fusion. **a** ORF3a blocked autophagosome–lysosome fusion, as shown by autophagy flux assays. HeLa cells with stable expression of mCherry-GFP-LC3 were observed by fluorescence confocal microscopy after expression of ORF3a. Scale bars, 0.25 μm. **b** ORF3a caused the accumulation of formed autophagosomes in HeLa cells. Control cells and ORF3a-expressing cells were analyzed by transmission electron microscopy. Arrows indicate the accumulated formed double-layered autophagosomes in cells. Scale bars, 10 μm.

To investigate whether ORF3a abolished the fusion of autophagosomes with lysosomes, autophagic flux assays were conducted by measuring the localization and intensity of the fusion protein mCherry-GFP-LC3. Before fusion, autophagosome-integrated mCherry-GFP-LC3 was evident with yellow puncta, indicating signaling by both mCherry and GFP; after fusion with lysosomes, mCherry-GFP-LC3 in the autophagosomes emitted only mCherry signal because the low pH inside lysosomes quenched the GFP fluorescence. Clearly, ORF3a expression caused the mCherry-GFP-LC3 puncta to emit both types of fluorescence, which indicated that ORF3a blocked the fusion of autophagosomes with lysosomes (Fig. 3a). This was further confirmed by electron microscopy (EM) of fully formed autophagosomes accumulating in ORF3a-expressing cells (Fig. 3b). Thus, SARS-CoV-2 ORF3a inhibited autophagic flux by blocking the fusion of autophagosomes with lysosomes.

### The transmembrane (TM) domain and C-terminus of SARS-CoV-2 ORF3a are necessary for blocking autophagy

**Fig. 4:**
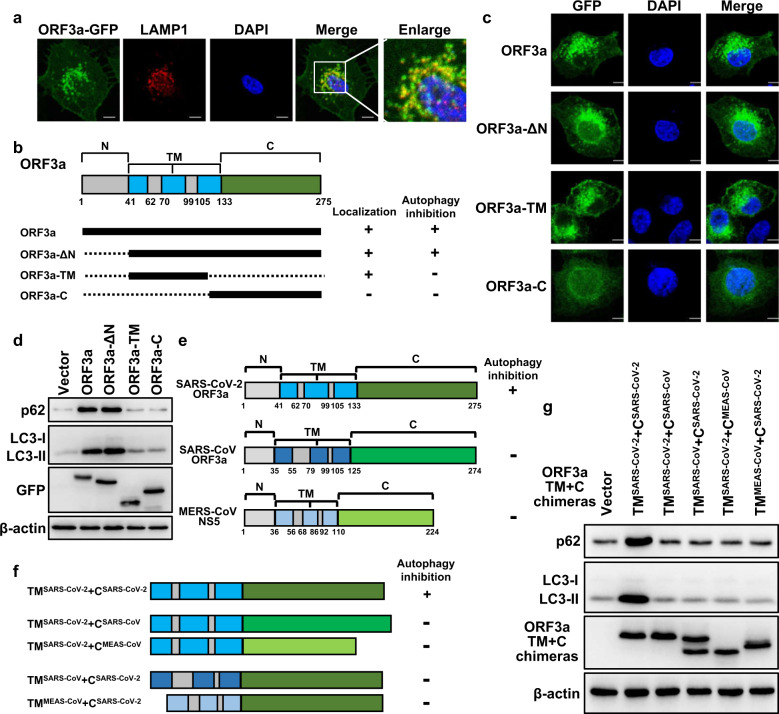
The TM domain and C-terminus are important for ORF3a-mediated blockade of autophagy. **a** ORF3a colocalized with lysosome. ORF3a-GFP was expressed in HeLa cells, and lysosomes were visualized by immunofluorescence with an anti-LAMP-1 antibody. Scale bars, 0.5 μm. **b** Schematic representation of ORF3a and the indicated truncated mutant used to determine the cellular localization and function of ORF3a in autophagy. **c** Fluorescence of GFP-tagged ORF3a and the indicated truncated mutants in HeLa cells. Scale bars, 0.25 μm. **d** ORF3a and the indicated truncated mutants were expressed in HeLa cells, and their effect on autophagy was analyzed by detecting the protein levels of p62 and LC3. **e** Schematic representation of the protein domains of ORF3a homologs in β-coronaviruses (SARS-CoV-2, SARS-CoV, and MERS-CoV). **f** Schematic representation of chimeras of the SARS-CoV-2 ORF3a TM domain with a C-terminus from an ORF3a homolog or the SARS-CoV-2 ORF3a C-terminus with a TM domain from an ORF3a homolog. **g** SARS-CoV-2 ORF3a TM + C-terminal truncated mutant or the indicated chimeras were expressed in HeLa cells, and their effect on autophagy was analyzed by measuring the protein levels of p62 and LC3-II.

We then sought to determine the underlying mechanism by which ORF3a blocks autophagosome–lysosome fusion. Interestingly, we found that ORF3a showed a puncta-like distribution in transfected cells, and some ORF3a was colocalized with LAMP1, a lysosome membrane marker protein (Fig. 4a). ORF3a was shown to have TM domains, an N-terminal region, and a C-terminal region (Fig. 4b). To test whether the special localization of ORF3a was important for its function in autophagy, different truncated ORF3a proteins were constructed and tested for their function in autophagy (Fig. 4b). The deletion of the N-terminus had no influence on either ORF3a localization or its function in blocking autophagy (Fig. 4b, d). The TM domain of ORF3a was distributed in cells similarly to wild-type ORF3a, while the C-terminus of ORF3a lost the puncta-localization feature (Fig. 4c). Neither the TM domain nor the C-terminus of ORF3a alone blocked autophagy (Fig. 4d). Next, we swapped the TM or C-terminus from ORF3a homologs with that in SARS-CoV-2 ORF3a. Among the three β-coronaviruses (SARS-CoV-2, SARS-CoV, and MERS-CoV), SARS-CoV-2 ORF3a uniquely inhibited autophagy (Supplementary Fig. S1c), although they contained similar TM and C-termini (Fig. 4e). The TM + C truncation of SARS-CoV-2 ORF3a was as potent as full-length ORF3a in autophagy inhibition (Fig. 4b, d). We constructed four chimeras: TM from SARS-CoV-2 ORF3a combined with C-terminus from SARS-CoV or MERS and the C-terminus from SARS-CoV-2 ORF3a combined with TM from SARS-CoV or MERS (Fig. 4f). These chimeras and TM + C-truncated SARS-CoV-2 ORF3a were tested to determine their effect on autophagy, and the results showed that none of these chimeras influenced the protein levels of p62 or LC3-II (Fig. 4f, g).

These results suggested that the special localization (probably to lysosomes) mediated by the TM domains, together with the C-terminus of ORF3a, are essential for its blockade of autophagy.

### ORF3a interacted with the HOPS complex component VPS39 and disrupted the RAB7–HOPS interaction

**Fig. 5:**
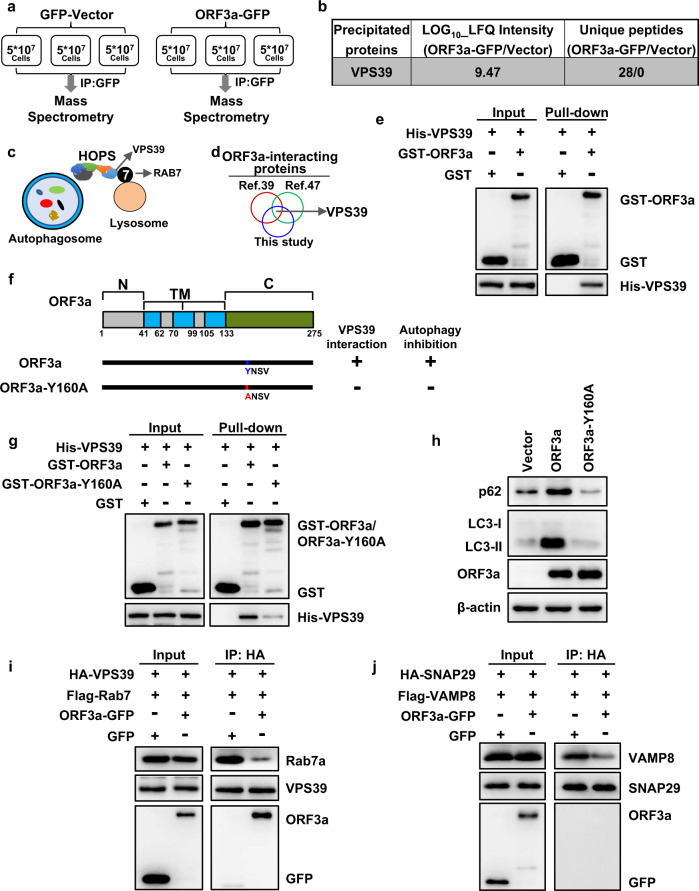
ORF3a interacted with the HOPS component VPS39 and prevented RAB7–VPS39 bundling. **a** Outline of co-immunoprecipitation and mass spectrometry experiments performed to identify ORF3a-interacting proteins in HeLa cells. **b** HOPS component VPS39 was the candidate among the ORF3a-interacting proteins identified by mass spectrometry at the highest confident level. **c** Scheme showing the function of HOPS–RAB7 in autophagosome–lysosome fusion. **d** Two experimental studies on ORF3a-interacting proteins and our study simultaneously identified VPS39. **e** ORF3a directly interacted with VPS39. GST, GST-ORF3a, or His-VPS39 were expressed in bacteria. The purified proteins were used for GST pull-down assays and subsequent western blot analysis. **f** Schematic representation of ORF3a and the indicated Y160A mutant used to verify the VPS39 interaction and function in autophagy. **g** The Y160A mutation in ORF3a dramatically reduced the interaction between ORF3a and VPS39. GST, GST-ORF3a, the GST-ORF3a-Y160A mutant or His-VPS39 were expressed in bacteria. Purified proteins were used for GST pull-down assays and subsequent western blot analysis. **h** The Y160A mutation in ORF3a abolished its function in blocking autophagy. **i** ORF3a prevented the RAB7 interaction with VPS39. The RAB7–VPS39 interaction was detected by co-immunoprecipitation assays with or without ORF3a expression. **j** ORF3a disrupted the assembly of SNARE. The interaction between SNARE components SNAP29 and VAMP8 was detected by co-immunoprecipitation assays with or without expression of ORF3a.

The lysosomal localization of ORF3a was in line with its effect on blocking autophagosome–lysosome fusion. To clarify how ORF3a blocks autophagosome–lysosome fusion, we analyzed the interacting proteins of ORF3a though immunoprecipitation followed by mass spectrometry analysis (the scheme is shown in Fig. 5a). We found that the strongest interacting protein of ORF3a was VPS39 (Fig. 5b), a component of the HOPS complex. The HOPS complex together with GTPase RAB7 is critical for tethering autophagosomes with lysosomes followed by fusion of the membrane bilayers^24,43–46^ (schemed in Fig. 5c). We were interested in the ORF3a–VPS39 interaction since the role of VPS39 in vesicle fusion is in line with the function of ORF3a in blocking autophagosome–lysosome fusion. Furthermore, VPS39 was found to interact with ORF3a in two contemporaneous experimental SARS-CoV-2 virus–host interactome studies^39,47^ in addition to our results (Fig. 5d). We speculated that the binding of VPS39 may be the mechanism by which ORF3a functions in blocking autophagy. The interaction between VPS39 and ORF3a was confirmed by co-immunoprecipitation assays (Supplementary Fig. S2a). This interaction was demonstrated as directly shown by GST pull-down assays (Fig. 5e). As shown above, the C-terminus of ORF3a was important for its function in blocking autophagy (Fig. 4b–d) and the YXXΦ motif (160-YNSV-163) in C-terminus was found to be important for apoptosis induction in host cells^48^. We then tested whether this motif was important for ORF3a function in autophagy and its interaction with VPS39. A point mutation in ORF3a (Y160A) abolished its interaction with VPS39 and abrogated its function in autophagy (Fig. 5f–h and Supplementary Fig. S2b). These results supported the hypothesis that the interaction of ORF3a with VPS39 blocked autophagy. Since RAB7 binds HOPS by interacting with VPS39 (the scheme is shown in Fig. 5c), we sought to determine whether ORF3a can affect the RAB7–VPS39 interaction. The results showed that ORF3a expression dramatically reduced the interaction between RAB7 and VPS39 (Fig. 5i). Furthermore, the assembly of the SNARE complex may also be disrupted by ORF3a, as shown by the disturbed interaction between SNARE components SNAP29 and VAMP8 in the presence of ORF3a (Fig. 5j and Supplementary Fig. S2c). This outcome is logical because the RAB7–HOPS interaction is important for the subsequent assembly of the SNARE complex^24,43–45,49,50^. Thus, ORF3a sequestration of VPS39 abolished the VPS39–RAB7 interaction and caused further failure of SNARE assembly.

**Fig. 6:**
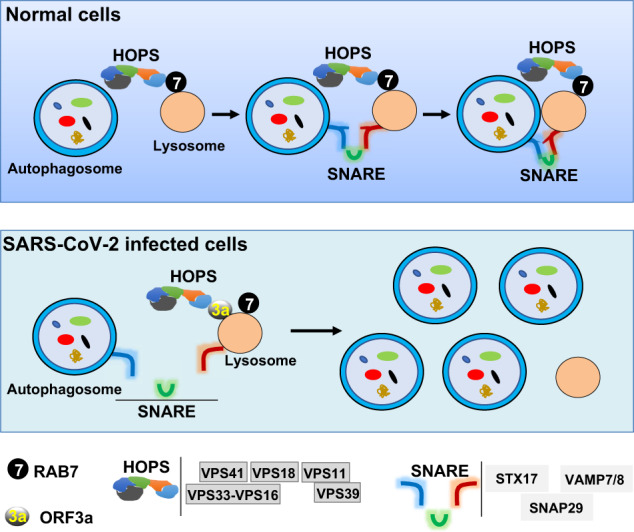
Schematic diagram of the function of SARS-CoV-2 ORF3a in blocking autophagosome–lysosome fusion. Upper, after double-layered autophagosomes are formed, they fuse with lysosomes for final degradation of their cargoes. GTPase RAB7 recruits the tethering HOPS complex, acting as a bridge to bring the two compartments together. These tethering HOPS complexes, in turn, help SNAREs physically drive the fusion of opposing lipid bilayers. Lower, when host cells are subjected to SARS-CoV-2 infection, ORF3a is expressed and interacts with VPS39. The ORF3a–VPS39 interaction sequesters VPS39, preventing its interaction with RAB7 and resulting in failure of HOPS–RAB7 assembly and blockage of autophagosome–lysosome fusion, which eventually leads to accumulation of autophagosomes.

In summary, we propose a potential mechanism by which ORF3a blocks autophagic flux. After autophagosomes are formed, they fuse with lysosomes for the final degradation of their contents. The GTPase RAB7 localizes to lysosome membranes and recruits tethering HOPS complexes to bring the lysosome and autophagosome compartments together. The HOPS complex, in turn, facilitates SNARE assembly, and SNARE complex physically drives the fusion of the opposing lipid bilayers (Fig. 6, upper). When host cells were subjected to SARS-CoV-2 infection, viral ORF3a was expressed and interacted with VPS39. The ORF3a–VPS39 interaction sequestered VPS39, preventing the VPS39–RAB7 interaction, resulting in failure of the HOPS–RAB7 and SNARE assemblies and thus blocked autophagosome–lysosome fusion (Fig. 6, lower).

## Discussion

Discovering the cytopathic effects of SARS-CoV-2 will greatly aid in developing novel therapeutics to treat COVID-19. As an intracellular degradation process, autophagy in host cells is an innate antiviral immune response to pathogen infection, including viral infection. Escape from autophagic clearance is important for virus survival and replication in host cells, especially considering that infection by viruses, including coronaviruses, triggers autophagy to battle against pathogens^51,52^. It is reasonably speculated that some viruses have evolved to remodel the autophagic process for their own benefit during replication^53,54^. In our study, we systematically analyzed SARS-CoV-2 protein effects on autophagy and demonstrated a mechanism by which viral ORF3a inhibits autophagosome–lysosome fusion: ORF3a disrupts the assembly of the RAB7–HOPS fusion machinery (Fig. 6).

One limitation of this study is that ectopically expressed ORF3a, not the SARS-CoV-2 virus, was tested for its effect on autophagy. It is important to show the effect of ORF3a on blocking autophagic flux and inducing incomplete autophagy under true SARS-CoV-2 infection conditions. Autophagy inhibition caused by SARS-CoV-2 infection was recently shown in three independent studies^41,55,56^. In these three studies, SARS-CoV-2 viral infection in NCI-H1299 cells, Vero FM cells^41^, and HeLa cells expressing human ACE2^55,56^ inhibited autophagic activity by blocking autophagosome–lysosome fusion. Considering our observation that, compared to other viral proteins, ORF3a caused the most effective inhibition of autophagy (Fig. 1b), it is speculated that ORF3a expressed during SARS-CoV-2 infection mediates the inhibition of autophagy in host cells.

One recent study showing ORF3a regulating autophagy, by Miao et al.^55^, is similar to our work presented here. Miao et al.^55^ found that SARS-CoV-2 ORF3a inhibits autophagy by blocking autophagosome–lysosome fusion. Mechanistically, they clarified that the ORF3a interaction with VPS39 disrupts the assembly of the HOPS and SNARE complexes^55^. In addition to the similar conclusions reached by Miao et al. and us, one superior point of our work is the finding that the C-terminal region, specifically the cytoplasmic YNSV motif, was essential for VPS39-binding and for ORF3a inhibition of autophagy (Fig. 5g, h). In addition, although both works analyzed the intracellular localization of ORF3a, our work clarified that the TM domain of ORF3a was important for ORF3a localization and its autophagy-inhibiting function (Fig. 4c, d). Combined with the observation that the N-terminus of ORF3a is not necessary for its localization and autophagy-inhibiting function (Fig. 4b–d), our work here provides information on the involvement and respective roles of each region/domain in ORF3a in regulating autophagy. Both our findings and those of Miao et al. show that ORF3a disrupts the assembly of the SNARE complex; additionally, our work showed that ORF3a prohibits the HOPS–Rab7 interaction, and Miao et al.^55^ showed in detail that ORF3a prohibits the assembly of the HOPS complex itself. We speculated that ORF3a-binding with VPS39 decomposes the HOPS complex, which leads to failure HOPS–Rab7 bundle formation, which is important for autophagosome–lysosome fusion^46^. In addition, Miao et al.^55^ showed that ORF3a weakly impairs the functions of lysosomes and that knocking down O-GlcNAc transferase OGT restores normal autophagic flux in ORF3a-expressing cells; this information was not included in our work here.

This role of ORF3a may protect SARS-CoV-2 from being cleared by autophagy in host cells. Many viruses, including coronaviruses, are known to rely on the formation of autophagosome-like viral double-membrane vesicles (DMVs) as platforms for optimal replication^52,57–62^. Although not fully understood, the host autophagic machinery is involved in the formation of DMVs^63,64^. Considering the similarity between DMVs and autophagosomes, ORF3a disruption of RAB7–HOPS may prevent DMVs from fusing with lysosomes. Intriguingly, two other coronaviruses, SARS-CoV and MERS-CoV, were found to block autophagosome–lysosome fusion through virus coded membrane-associated papain-like protease PLP2^33,34,65–67^. Therefore, the inhibition of autophagosome–lysosome fusion may be a conserved mechanism for these highly pathogenic coronaviruses that have caused disease pandemics in recent years. The inhibition of autophagy by SARS-CoV-2 may explain why the lysosomotropic agent CQ does not block SARS-CoV-2 infection^68,69^. The role of CQ in blocking autophagosome fusion and neutralizing the pH of lysosomes is partially redundant with the ORF3a function discovered here. Our findings provide clues for potential drug development targeting the autophagic pathway for the treatment of COVID-19. In support of this concept, recent studies demonstrated that autophagy induction by calorie restriction or the utility of three different autophagy-inducing drugs, spermidine, MK02206, and niclosamide, can restrict SARS-CoV-2 propagation^41,70^.

## Materials and methods

### Cell culture

Standard cell culture techniques were used. HeLa (ATCC, #CCL-2), HEK293T (ATCC, #CRL-11268), and A549 (ATCC, #CCL-185) cells were grown in DMEM (Invitrogen) supplemented with 10% FBS (FBS; Gibco, Life Technologies), 2 mM L-glutamine, and 100 U/ml penicillin-streptomycin in a humidified incubator at 37 °C with 5% CO_2_. For treatment of INK128 (5 μM), CQ (200 μM), cells were treated for 4 h before harvest. For starvation assay, cells were washed three times with PBS and then cultured in EBSS for 2 h.

### Plasmids and transfection

The SARS-CoV-2 expression plasmids were kindly provided by Dr. Nevan J. Krogan (University of California San Francisco, USA). All proteins encoded with exception of NSP3 and NSP16 were codon optimized and cloned into a mammalian expression vector pLVX-EF1alpha with a 2× Strep tag. ORF3a-GFP and its mutants were generated by cloning ORF3a into an in-house modified version of pLVX-EF1alpha-GFP vector. cDNAs encoding VPS39, SNAP29 were cloned into pLVX-puro vector. cDNAs encoding RAB7, STX17, and VAMP8 were cloned into pFLAG-CMV2 vector. cDNAs of SARS-CoV ORF3, MEAS-CoV ORF5, HCoV-229E NS5, and IBV ORF3a purchased from General Biosystems (General Biosystems, Anhui, China) and then cloned into pLVX-EF1alpha-GFP-puro vector. Transfection reagent, Lipofectamine 2000 (Invitrogen), was purchased from Invitrogen and used according to the manufacturer’s protocol.

### Protein purification

The DNAs of SARS-CoV-2 ORF3a and its mutation ORF3a-Y160A were subcloned into pGEX-4T1. The DNA of VPS39 was subcloned into pET28-a. Recombinant proteins were expressed in *Escherichia coli* BL21-CodonPlus (DE3) cultured in LB medium, after induction with 0.2 mM IPTG overnight at 16 °C, the bacteria were pelleted by centrifugation at 4000× *g* for 10 min. The pellets were lysed in 25 mM Tris-HCl pH 8.0, 150 mM NaCl, 0.5 mM TCEP-HCl or 1 mM DTT, 1 mM PMSF, 0.8 µM Aprotinin, 1 µM pepstatin, and 10 µM Leupeptin by FrenchPress. The lysates were centrifuged at 25,000× *g* for 30 min at 4 °C, and then purified using Ni-NTA Sefinose (TM) Resin (for His-VPS39, BBI) and GST-Sefinose (TM) Resin (for GST-ORF3a and GST-ORF3a-Y160A, BBI). Target proteins were further purified on a Superose 10/300 GL column equilibrated with 25 mM Tris-HCl pH 8.0, 150 mM NaCl, and 2 mM DTT. The peak fractions were pooled and flash-frozen in liquid nitrogen for storage. All purified proteins were assessed by running and staining in SDS-PAGE gels.

### GST pull-down assays

For the pull-down assay, 30 μg GST, GST-ORF3a, and GST-ORF3a-Y160A proteins were incubated with 30 μL glutathione Sepharose beads in incubation buffer (150 mM NaCl, 25 mM Tris-HCl pH 8.0, 0.1% NP40, and 5% glycerol) at 4 °C for 2 h, respectively. The beads were washed three times with the incubation buffer and then incubated with 30 μg of His-VPS39 at 4 °C for 3 h. After rinsed with incubation buffer for five times, the resin was resuspended in loading buffer for WB analysis.

### SARS-CoV-2 infection in Calu-3 cells

A clinical isolate nCoV-2019BetaCoV/Wuhan/WIV04/2019 was propagated in Vero E6 cells. All the infection experiments were performed in a biosafety level-3 laboratory. Briefly, Calu-3 cells were infected with SARS-CoV-2 at a multiplicity of infection of 0.05. Viral N protein and ORF3a expression in infected cells was analyzed by western blot using primary antibodies anti-NP (Sino biological, 40143-MM08) and anti-ORF3a (Bioworld Technology, NCP0017) at 48 h postinoculation.

### Western blotting and antibodies

Cell lysates were collected in lysis buffer and quantified by Bradford protein assay (Thermo Scientific), heated to 95 °C for 10 min. Proteins were separated by SDS-PAGE and transferred to PVDF membrane (Bio-Rad), and all western blotting steps were performed in TBST containing 5% milk. All membranes were probed overnight with indicated antibodies at 4 °C. Appropriate HRP-conjugated secondary antibodies were incubated on membranes and bands were developed with ECL reagent (Millipore) and serial time exposure with signal saturation avoidance (saturated signal will be labeled with red color in ChemiDoc MP Imaging System, Bio-Rad). Monoclonal antibodies against HA-epitope (F-7) and GFP (B-2) were purchased from Santa Cruz Biotechnology. Monoclonal anti-FLAG (F3165) was from Sigma. Mouse anti Strep II-Tag mAb (AE066) and monoclonal anti-β-actin (AC026) were from ABclonal. Monoclonal LC3 antibody (4108) was from Cell Signaling Technology. Monoclonal anti-p62 (109102) was from Abcam. Mouse monoclonal antibodies anti-GST (66001) and anti-His (66005) were from Proteintech.

### Fluorescence and immune-fluorescence assays

For immunofluorescence microscopy, cells were seeded onto precision cover glass. Cells were then fixed with 4% paraformaldehyde (10 min at room temperature) and permeabilization in 0.1% Triton X-100 (20 min on ice). Following block with 1% BSA, cells were incubated with primary antibodies followed by incubation with Alexa Fluor-conjugated secondary antibodies. Finally, cells were equilibrated in PBS, stained for DAPI (0.5 μg/ml). Images were acquired on a Zeiss LSM 880 microscopes. Representative images of at least three independent replicates are shown.

### Co-immunoprecipitation assays

For binding studies involving co-IP, cultured HeLa or A549 cells were transfected with the appropriate plasmids with Lipofectamine 2000 (Invitrogen) according to the manufacturer’s protocol; HEK293T cells were transfected with the appropriate plasmids by standard calcium phosphate precipitation method. Cells were harvested and lysed with lysis buffer (50 mM Tris, 150 mM NaCl, 10% glycerol, protease inhibitors) for 30 min. The supernatants were collected by centrifugation at 5000 rpm for 5 min at 4 °C and precleared by incubated with GFP-Trap agarose beads (Chromotek) or HA-Beads (Roche) for 2 h at 4 °C with rotation. After centrifugation, IPs were followed by stringent washing steps to remove nonspecific background binding to the beads. Then the beads were boiled at 95 °C for 10 min and analyzed by western blot.

### Transmission electron microscopy

HeLa cells were washed three times for 15 min with 0.1 M phosphate buffer, and then fixed in 2% aqueous osmium tetraoxide for 1 h followed by washing three times each for 15 min with deionized water. Samples were then dyed with 2% uranyl acetate for 30 min, and dehydrated through graded alcohols (50%–100%) and 100% acetone each for 15 min. After that, samples were embedded in EPON 812 resin and cured for 24 h at 37 °C, 45 °C, 60 °C, respectively. Ultra-thin (70 nm) sections were obtained by ultra-thin slicer machine and stained with 2% uranyl acetate and 0.3% lead citrate. EM images of the samples were taken using Tecnai G2 Spirit transmission electron microscope (FEI Company). Representative images of at least three independent replicated experiments are shown.

### Statistical analysis

All experiments were independently repeated at least three times with consistent conclusions and representative results were shown. Values are expressed as means ± SD at least three independent experiments. The significance of the variability between different groups was determined by two-way ANOVA tests of variance using the GraphPad Prism software (version 6.0). *P* < 0.05 was considered statistically significant, and *P* > 0.05 was considered statistically nonsignificant.

## Supplementary information

Supplementary Figure S1

Supplementary Figure S2
