# Supplementary material for: The SARS-CoV-2 protein ORF3a inhibits fusion of autophagosomes with lysosomes
Source: Cell Discov. 2021 May 4;7:31. doi: 10.1038/s41421-021-00268-z (PMC8096138; doi:10.1038/s41421-021-00268-z)
Supplement: Supplementary file 1 — Supplementary Figure S1 [file 41421_2021_268_MOESM1_ESM.pdf]

Fig. S1

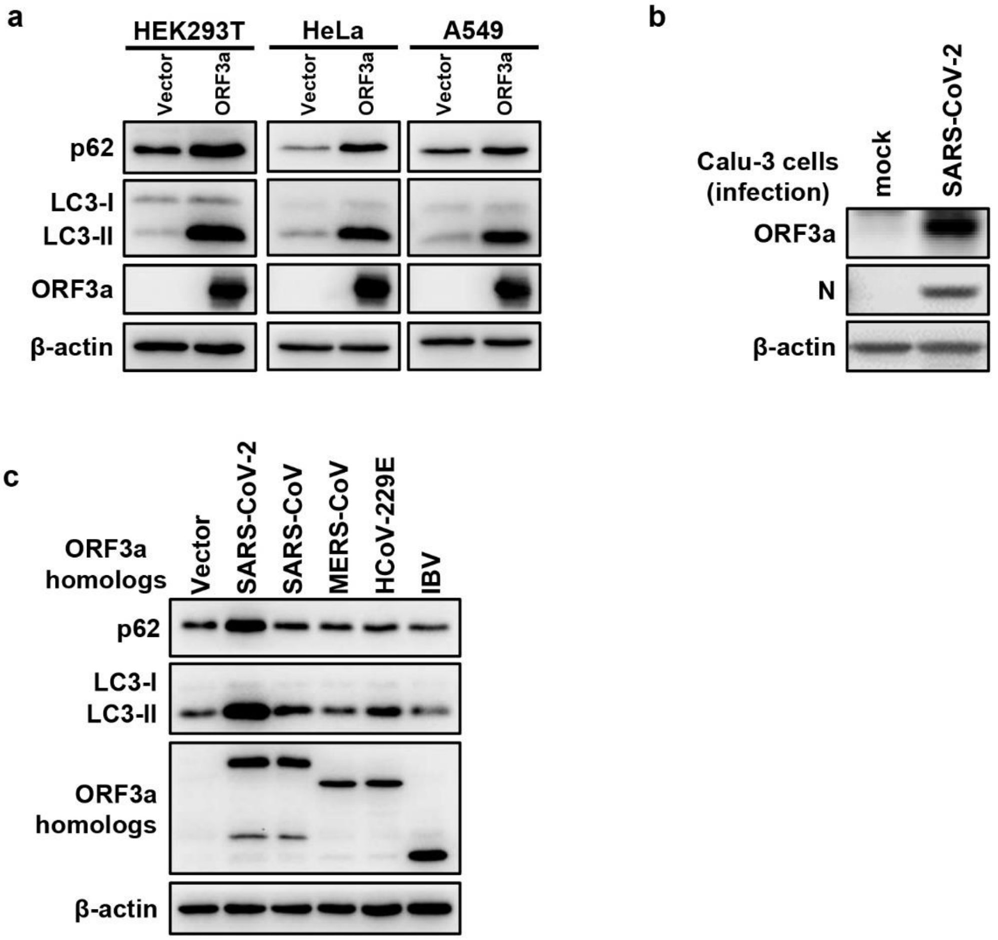

Fig. S1 SARS-CoV-2 ORF3a specifically inhibited autophagy.

a. SARS-CoV-2 ORF3a was expressed in different cell lines, and its effect on autophagy was confirmed by measuring the protein levels of p62 and LC3-II.

b. SARS-CoV-2 ORF3a was expressed during viral infection of human cells. Human Calu-3 cells were infected with SARS-CoV-2 virus obtained by clinical isolation, and 48h after infection the expression of ORF3a and structural N protein was measured.

c. SARS-CoV-2 ORF3a specifically inhibited autophagy. ORF3a homologs from α-CoV (HCoV-229E), β-CoV (SARS-CoV-2, SARS-CoV, MERS-CoV) and γ-CoV (IBV) were expressed in HeLa cells, and their effect on autophagy was analyzed by measuring the protein levels of p62 and LC3-II.
