# Supplementary material for: The SARS-CoV-2 protein ORF3a inhibits fusion of autophagosomes with lysosomes
Source: Cell Discov. 2021 May 4;7:31. doi: 10.1038/s41421-021-00268-z (PMC8096138; doi:10.1038/s41421-021-00268-z)
Supplement: Supplementary file 2 — Supplementary Figure S2 [file 41421_2021_268_MOESM2_ESM.pdf]

Fig. S2

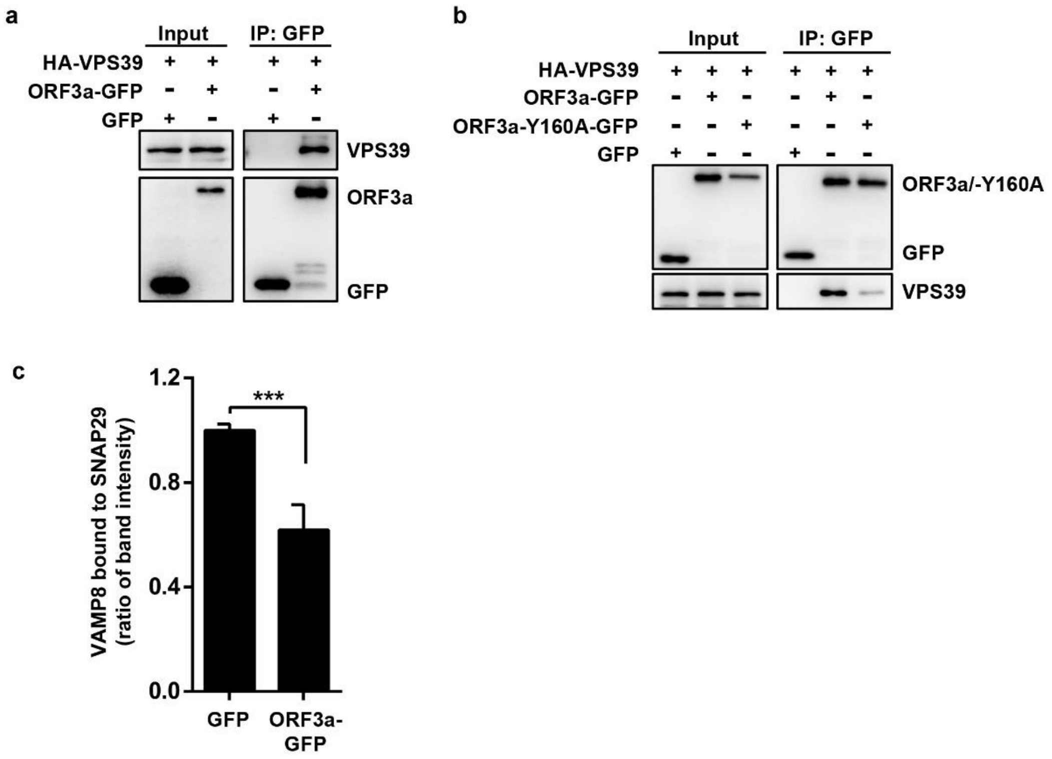

Fig. S2 ORF3a interacted with HOPS component VPS39.

a. ORF3a was found to interact with VPS39, as shown by the co-IP assays.

b. The Y160A mutation in ORF3a dramatically reduced the interaction between ORF3a and VPS39, as shown by coimmunoprecipitation assay.

c. Quantification of the amounts of VAMP8 bound by SNAP29 in the presence of GFP or ORF3a-GFP. The band intensity in the western blots (IP panels) of VAMP8 was compared to that of SNAP29 in Fig. 5j. The results from three completed experiments were analyzed. ***: $P<0.001$.
